# Western Ophthalmologic and Oto-Laryngo-Logic Association

**Published:** 1900-05

**Authors:** 


### *SOCIETY REPORTS.*

### WESTERN OPHTHALMOLOGIC AND OTO-LARYNGO-LOGIC ASSOCIATION.

The Western Ophthalmologic and Oto-laryngologic Association held its fifth annual meeting at St. Louis on the 5th, 6th and 7th of April. The president, Dr. W. Scheppergrell of New Orleans, opened the meeting with a paper on "The Rise of Specialism," in which he disproved the oft-repeated charge that specialisms in medicine are modern innovations. He cited historical data dating several centuries before Christ in which distinct references were made to specialists of the eyes, stomach and the head. The essayist commended specialists in medicine, as they promote more detailed study and thereby lead to higher medical attainments.

J. W. Bullard, M.D., of Pawnee City, Neb., read a paper on "Two Classes of Eye Cases that Give Me a Great Deal of Trouble." Chief among them were those in which irritation and dryness of the conjunctiva persisted in spite of every attempt at refraction which had been made.

Edwin Pynchon, M.D., of Chicago, "Slight Irregularities of the Nasal Septum." The author advocated the removal or correction of slight irregularities of the septum when there were disturbances of the nasal functions on account of their presence. If later and larger development justified their removal, the author thought their early removal was justified on the grounds of "A stitch in time saves nine."

C. R. Holmes, M.D., Cincinnati, "Foreign Bodies of the Orbit, with Report of Cases." About seventy cases were compiled from the literature by the author, and three additional ones reported by him. The most interesting and unique case was one reported by the author. It consisted of a knife blade about 1½ inches long, which had been in the orbit thirty-two years without causing much inconvenience. It was imbedded in a fibrous capsule and was but slightly rusted.

B. E. Freyer, M.D., Kansas City, "Report of a Case of Railway Trauma of the Eye, with the Report of a Case and its Legal Aspect."

M. A. Goldstein, M.D., St. Louis, "Presentation of Cases: (*a*) Primary Tuberculosis of the Ear; (*b*) Primary Tuberculosis of the Larynx." (*a*) The case had been operated on some years previously and had a recurrence some months ago, at which time Dr. Goldstein did the Schwartz operation. Bad symptoms developed a few weeks ago and he did the radical operation, since which time the patient is doing well. (*b*) The second case was one of probable primary tuberculosis of the larynx, which came under the observation of the author about one year ago. At that time he was in a very serious condition; death seemed but a matter of a few weeks or months. The patient was greatly emaciated, and in response to the treatment administered had gained a fair degree of health, being able to attend to business. The diagnosis in the case is somewhat doubtful, but the author having excluded lues and malignant growth, has made the diagnosis of primary laryngeal tuberculosis. Tubercle bacilli are absent and the tissue has not been examined microscopically.

Dr. Wm. L. Ballenger, of Chicago, "The Physiologic Tests of the Organs of Hearing as Aids in the Differential Diagnosis of Lesions of the Ear." The author advocated the physiologic tests of the ear, including the range of hearing, as tested with the tuning fork, the Galton whistle, the Webber experiment, the Rinne experiment, Schwaback and Bing tests, as important aids in the differential diagnosis of the lesions of the ear. They are of special importance in determining the location of the lesion. He suggested that in a general way the deeper the structure involved, the more pronounced the disturbance of hearing and the less probability of a cure. The tests were therefore recommended more for the purpose of aiding the surgeon in giving a correct diagnosis than for the purpose of aiding him in the treatment, which is often unsuccessful. Six cases were cited illustrating lesions of different kinds in the middle ear and labyrinth in which the tests were used for the purpose of differentiating them. He recommended that the tests be made in all cases of ear diseases in which there was marked deafness and tinnitus, both before and after inflation of the tympanum. If this point is neglected the diagnosis may not be properly made. While the physiologic tests are not absolute guides to a correct diagnosis, they are, together with all the other means of diagnosis, the most correct at the command of the aural surgeon, and, therefore, should be invariably used.

O. J. Stein, M.D., Chicago, "Symmetrical Osteoma of the Nose; Report of a Case." The author reported a very rare case of symmetrical or double osteoma of the nose, occluding the nasal chambers and extending to either side for a considerable distance, whereby the patient was given the typical frog-face appearance. Osteoma upon one side is rather common. This case was presented on account of its unique type and was reported with a number of other cases collected from the literature. No attempt was made to correct the deformity, as the patient is well advanced with tuberculosis, several other members of the family having died with the same disease.

Jno. J. Kyle, M.D., Indianapolis, "The Sympathetic Inflammation and Sympathetic Irritation of the Eye." The author made an interesting review of the subject presented, in which he advocated the usual classical treatment.

Adolph Alt, M.D., St. Louis, "Studies Concerning the Anatomy of the Eyelids, Especially Their Glands (with lantern slides)." The purpose of the author was to report the result of an extensive examination made of the tissues of the eyelids, in which he had found mucous glands located in positions where they were not usually found. He also stated that in all his examinations, with one exception, the torsal cartilages of the eyelids were not true cartilaginous tissue.

H. W. Loeb, M.D., St. Louis, "Presentation of Specimen of 107 Polypi Removed at One Sitting." This case was unique, not so much on account of the great number of polypi removed from the nose as from the fact that they were removed at a single sitting. They were uniformly pedunculated and varied greatly in size. They were removed with an electro-cautery snare devised by the author.

J. O. Stillson, M.D., Indianapolis, "Removal of the Middle Turbinate for the Cure of Some Forms of Inveterate Eye Disease." The author read a very interesting paper on this subject, in which he reported his observations as to the relationship of nasal and eye diseases and the results he had obtained in allaying eye symptoms by the treatment of nasal conditions, more especially the removal of the turbinate.

Dr. Goldstein, as chairman of the local committee of arrangements, arranged for a museum of pathologic and anatomic specimens, which, while not large, was extremely interesting and marks a new departure in this society.

The officers elected for the ensuing year were as follows:

Dr. M. A. Goldstein of St. Louis, President.

Dr. Würdemann of St. Paul, 1st Vice-President.

Dr. C. R. Holmes of Cincinnati, 2d Vice-President.

Dr. Fayette C. Ewing, St. Louis, 3d Vice-President.

Dr. W. L. Dayton of Lincoln, Neb., Treasurer.

Dr. Wm. L. Ballenger of Chicago, Secretary.

Dr. C. R. Holmes was made chairman of the local committe of arrangements for the meeting to be held in Cincinnati next year.

Dr. Loeb of St. Louis, chairman of the membership committee.

---

### URIC ACID AS THE CAUSE of ASTHMA.

Continued observations make the uric acid explanation of the very large majority of all cases of asthma, especially those accompanied by nasal polypi, appear more and more rational, and more and more refer these polypi to a place among the symptoms, removing them from their widely assigned place as the cause of asthma. This statement is one of the greatest interest in the study of asthma, and at once suggests that polypoid degeneration of the ethmoidal bodies, in part or in whole, is in many cases the result of repeated uric acid irritation of these bodies. This statement will, I believe, some day be proven beyond question by the nasal microscopist. It is useless to look for the cause of a polypus in the growth itself; it must be sought for in the ethmoidal tissue from which it springs. Study of certain cases, supplemented by extended observation in a number of others, makes clear what might be expected—removal of nasal polypi no more cures asthma than would the removal of tophi about the joints of the fingers cure gout.—*Dr. John Dunn*, *Richmond*, *Va.*

